# Ginger extract inhibits LPS induced macrophage activation and function

**DOI:** 10.1186/1472-6882-8-1

**Published:** 2008-01-03

**Authors:** Sudipta Tripathi, David Bruch, Dilip S Kittur

**Affiliations:** 1 Dept of Surgery, SUNY Upstate Medical University, Syracuse, NY, 13210, USA

## Abstract

**Background:**

Macrophages play a dual role in host defence. They act as the first line of defence by mounting an inflammatory response to antigen exposure and also act as antigen presenting cells and initiate the adaptive immune response. They are also the primary infiltrating cells at the site of inflammation. Inhibition of macrophage activation is one of the possible approaches towards modulating inflammation. Both conventional and alternative approaches are being studied in this regard. Ginger, an herbal product with broad anti inflammatory actions, is used as an alternative medicine in a number of inflammatory conditions like rheumatic disorders. In the present study we examined the effect of ginger extract on macrophage activation in the presence of LPS stimulation.

**Methods:**

Murine peritoneal macrophages were stimulated by LPS in presence or absence of ginger extract and production of proinflammatory cytokines and chemokines were observed. We also studied the effect of ginger extract on the LPS induced expression of MHC II, B7.1, B7.2 and CD40 molecules. We also studied the antigen presenting function of ginger extract treated macrophages by primary mixed lymphocyte reaction.

**Results:**

We observed that ginger extract inhibited IL-12, TNF-α, IL-1β (pro inflammatory cytokines) and RANTES, MCP-1 (pro inflammatory chemokines) production in LPS stimulated macrophages. Ginger extract also down regulated the expression of B7.1, B7.2 and MHC class II molecules. In addition ginger extract negatively affected the antigen presenting function of macrophages and we observed a significant reduction in T cell proliferation in response to allostimulation, when ginger extract treated macrophages were used as APCs. A significant decrease in IFN-γ and IL-2 production by T cells in response to allostimulation was also observed.

**Conclusion:**

In conclusion ginger extract inhibits macrophage activation and APC function and indirectly inhibits T cell activation.

## Background

Macrophages are a major cell population of the innate immune system. They play an important role in mounting an inflammatory response, both in absence and presence of antigen, by secreting a number of cytokines and chemokines. These cytokines and chemokines influence the maturation and differentiation of the neighbouring cells of both the innate and adaptive immune system, which further enhances the inflammation. Other than being the first line of defence, macrophages also act as important accessory cells in the adaptive immune response.

Macrophages play a role in the activation of the adaptive immune system by behaving as antigen presenting cells (APCs), the most important outcome of macrophage activation and maturation. Activated macrophages express MHC class II molecules and costimulatory molecules like CD80, CD86 and CD40 and induce an effective T cell response in presence of an antigen-dependent inflammatory response. An effective T cell activation by macrophages requires MHC/T cell receptor interaction and costimulatory molecules on T cells and macrophages, which is supplemented by secretion of cytokines and chemokines by the macrophages.

In conditions like transplantation macrophages infiltrate the graft at an early stage and initiate an inflammatory response against the graft and also act as APCs thereby activating the T cell mediated graft rejection course [1]. In conditions like transplantation macrophages infiltrate the graft at an early stage and initiate an inflammatory response against the graft and also act as APCs thereby activating the T cell mediated graft rejection course [1]. These primary graft infiltrating cells are recruited from the circulation in response to the danger signal provided by the presence alloantigen. During this antigen dependent innate immune response macrophages promote inflammation and other biological functions like ischemia reperfusion injury which are associated with acute graft rejection [1]. It has also been reported that macrophages derived from peritoneal exudates act as more efficient APCs in rejecting allografts where HSP60 expression is high [2,3].

Ginger, a widely used anti-inflammatory herbal remedy is reported to inhibit macrophage activation and function [4,5]. It is used as an anti-inflammatory agent in diseases such as rheumatoid arthritis [1,6]. Kim *et al.*, reported the suppressive effects of ginger on reactive oxygen and nitrogen species generation and expression of inducible pro inflammatory genes [7]. Ginger is also reported to inhibit beta-amyloid peptide-induced cytokine and chemokine expression in cultured THP-1 monocytes [4] and suppress induction of chemokine expression in human synoviocytes [8,9].

We have previously reported the immunosuppressive effects of ginger on T cell proliferation [10,11]. We have also shown that 6-gingerol, one of the active components of ginger, is able to inhibit macrophage function selectively [12]. In the present study we determined the mechanism by which ginger extract inhibits T cell proliferation. We hypothesized that ginger extract inhibits T cell proliferation by inhibiting macrophage maturation and APC function. Our results indicate that ginger extract suppresses the antigen presentation function of macrophages by decreasing MHC class II molecule expression. It also decreases the costimulatory molecule expression, the second signal essential for T cell activation, and also inhibits the cytokine and chemokine production. Our results also show that ginger extract treated macrophages were unable to induce T cell activation leading to proliferation in presence of alloantigen stimulation.

## Methods

### Mice

Male and female C57Bl and Balb/C mice aged 6–8 weeks were purchased from The Jackson Laboratory. All mice were maintained in specific pathogen free animal facility at the SUNY Upstate Medical University.

### Isolation of peritoneal macrophages

Peritoneal exudates macrophages were harvested by peritoneal lavage from C57Bl mouse by i.p. injection of 10 ml sterile ice cold PBS (10 mM). The cells were washed with PBS and resuspended in RPMI 1640 supplemented with 10% FBS.

### Purification of murine T cells

Murine T cells were purified from C57Bl spleen using Murine T cell enrichment kit (SpinSep, Stem cell Technologies) according to manufacturer's instruction. In brief, 10^8 ^nucleated spleen cells were incubated for 30 min. with SpinSep enrichment cocktail followed by 20 min. incubation with SpinSep dense particle. The cells were next layered over SpinSep density medium and subjected to a density gradient centrifugation. Enriched cells were removed from the interface and washed and resuspended in RPMI 1640 supplemented with 10% FBS.

### Cell culture

Murine (C57Bl) peritoneal macrophages were cultured in RPMI 1640 supplemented with 10% FBS in 96 well flat bottomed plates at a concentration of 10^6^cells/ml. The cells were stimulated with LPS (100 ng/ml) in presence or absence of alcoholic ginger extract (1 μl/ml, Eclectic Institute) for 24 h. in a humidified 5% CO_2 _incubator. At the end of the incubation culture supernatants were collected and stored at -20°C for cytokine and chemokine assay.

### Mixed lymphocyte culture

C57Bl peritoneal macrophages (10^6^cells/ml) were treated with LPS in presence or absence of ginger extract for 24 h. in a humidified 5% CO_2 _incubator. Syngeneic T cells (10^6^cells/ml) purified from the splenocytes were added to the treated macrophages in presence of alloantigen stimulation. Gamma irradiated splenocytes (10^5^cells/ml) from Balb/C were used as the stimulator cells. After 7 days of incubation T cell proliferation and IL-2 and IFN-γ production was estimated. T cell proliferation was measured by non radioactive cell proliferation assay using MTS (CellTiter 96^® ^AQ_ueous_, Promega). IL-2 and IFN-γ production was assayed by ELISA (R&D Systems).

### Flow cytometry analysis

Mouse peritoneal macrophages were stimulated with LPS (100 ng/ml) in presence or absence of alcoholic ginger extract (1 μl/ml) for 24 h. in a humidified 5% CO_2 _incubator and were analyzed using B7.1-PE, B7.2-FITC, MHC II-APC mAb conjugates (eBiosciences) for costimulatory molecule and MHC II expression. Isotype controls were included as appropriate. The data were analysed using WinMDI software (TSRI Flow Cytometry Core Facility).

### Measurement of cytokines and chemokines by ELISA

Culture supernatants from ginger extract/LPS treated macrophages were assayed for pro-inflammatory cytokines namely TNF-α, IL-12 and IL-1β and chemokines namely RANTES, MCP-1, IP-10 by ELISA using assay kits from R&D Systems according to manufacturer's instructions. The supernatants from MLC were assayed for IL-2 and IFN-γ by ELISA (R&D Systems)

### Statistical analysis

All experiments were done in triplicates. "n" represents the number of mice used for each experiment. Data is represented as the arithmetic mean ± standard deviation. Comparison between the groups was analyzed using Student's t test. The accepted level of significance was p < 0.05.

## Results

### Ginger extract inhibits LPS induced macrophage activation

**Figure 1:**
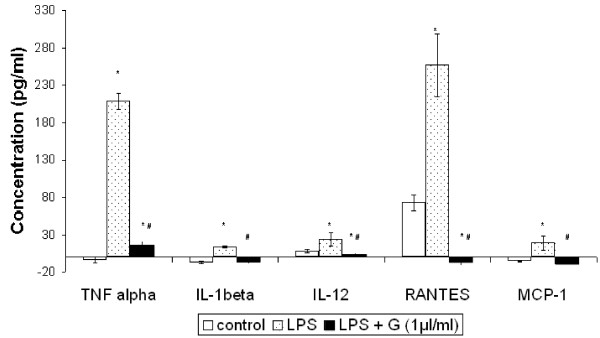
**Effect of alcoholic ginger extract on cytokine and chemokine release by LPS stimulated macrophages**. Peritoneal macrophages from C57Bl/6 mouse were isolated and stimulated with 100 ng/ml LPS in the absence or presence of ginger extract for 24 h. The cell supernatants were collected and cytokine and chemokine production was determined by ELISA. C- Control, LPS- LPS stimulated, LPS + G- LPS stimulation in the presence of ginger extract. Data represent mean ± S.D (n = 9). * *p *≤ 0.05 in comparison to LPS stimulated macrophages. # *p *≤ 0.05 in comparison to LPS.

LPS induced activation resulted in increased production of TNF-α (209.01 pg/ml), IL-12 (23.75 pg/ml) and IL-1β (13.9 pg/ml) by murine peritoneal macrophages. We observed a significant decrease in the LPS induced production of the above mentioned cytokines in presence of ginger extract treatment. A decrease of almost 10 fold in TNF-α production was observed by macrophages treated with ginger extract in presence of LPS stimulation (15.9 pg/ml) in comparison to macrophages stimulated with only LPS. In presence of ginger extract, LPS induced IL-1β production was completely inhibited. LPS induced IL-12 production was also decreased in presence of ginger extract treatment. Figure 1 shows the effect of ginger extract (1 μl/ml) on the LPS induced production of TNF-α, IL-12 and IL-1β by murine peritoneal macrophages.

In addition we also studied the effect of ginger extract on chemokine production namely, RANTES and MCP-1. We observed a complete inhibition of the LPS induced production of RANTES and MCP-1 by ginger extract as shown in Figure 1. LPS activation increased the production of RANTES from 72.96 pg/ml (control) to 256.9 pg/ml (LPS). No RANTES production was detected by LPS induced macrophages in presence of ginger extract. A similar effect of ginger extract on MCP-1 production was also observed (Control- not detected, LPS- 18.92 pg/ml, LPS+G- not detected).

### Ginger extract suppresses costimulatory molecule expression on LPS stimulated macrophages

**Figure 2:**
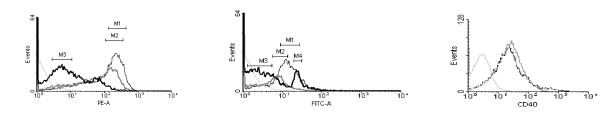
**Effect of alcoholic ginger extract on costimulatory molecule expression on murine peritoneal macrophage**. Peritoneal macrophages from C57Bl/6 mouse were isolated and stimulated with 100 ng/ml LPS in presence or absence of ginger extract for 24 h. The cells were harvested and stained with fluorophore conjugated mAb against B7.1 (**A**), B7.2 (**B**) and CD40 (**C**) and analyzed in LSR II flow cytometer. Data are representative result of six replicates.

LPS stimulation results in macrophage activation and increased production of pro inflammatory cytokines. The activity of LPS is further augmented by the key costimulatory pathway (interaction between CD28 and CD80-CD86) for T cell activation. Signalling through CD28 by CD80-CD86 is essential for the retained activity of LPS. However, LPS alone does not increase the expression of CD80-CD86 (Foss et al., 1999b; Foss et al.). Therefore we further studied the effect of ginger extract on the expression of CD80, CD86 and CD40 on murine peritoneal macrophages in presence of LPS stimulation. We observed a decrease in CD80 and CD86 expression on LPS stimulated macrophages in presence of ginger extract treatment. However, ginger extract had no such inhibitory effect on CD 40 expression. The histograms in Figure 2A depict the expression CD80. Region M1 and M2 represent control macrophages and LPS stimulated macrophages respectively. M3 region denotes CD80 expression on macrophages treated with ginger extract and LPS. CD86 and CD40 expressions are shown in Figure 2B and 2C respectively.

### Ginger extract decreases MHC class II expression on LPS stimulated macrophages

**Figure 3:**
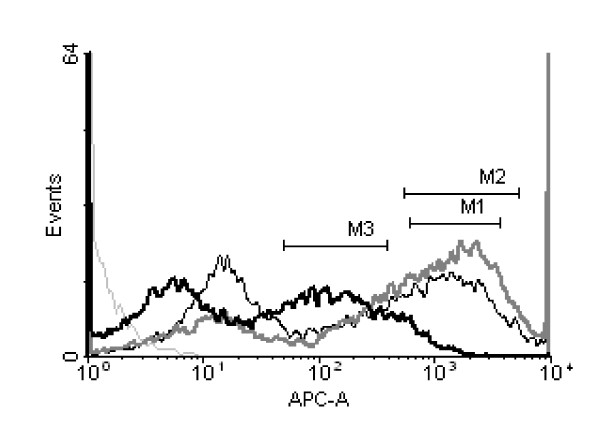
**Effect of alcoholic ginger extract on MHC class II molecule expression on murine peritoneal macrophages**. Peritoneal macrophages from C57Bl/6 mouse were isolated and stimulated with 100 ng/ml LPS in presence or absence of ginger extract for 24 h. The cells were harvested and stained with fluorophore conjugated mAb against MHC II and analyzed in LSR II flow cytometer. Data are representative result of six replicates.

Activated macrophages are mobilized in presence of an inflammatory response to behave as antigen presenting cells. The APC function of the macrophages is regulated by a controlled expression of the MHC class II molecule. We have previously observed that ginger extract decreases the expression of costimulatory molecules on macrophages. In continuation we examined the effect ginger extract has on the expression of MHC class II molecule in peritoneal macrophages. We observed that ginger extract decrease the expression of MHC class II molecule in LPS stimulated macrophages as shown in Figure 3. A decrease in fluorescence intensity indicating a low expression level of MHC II was observed in case of ginger extract treated macrophages in presence of LPS treatment, in comparison to that of control and LPS treated macrophages.

### Ginger extract treated macrophage restrict alloantigen induced T cell proliferation

**Figure 4:**
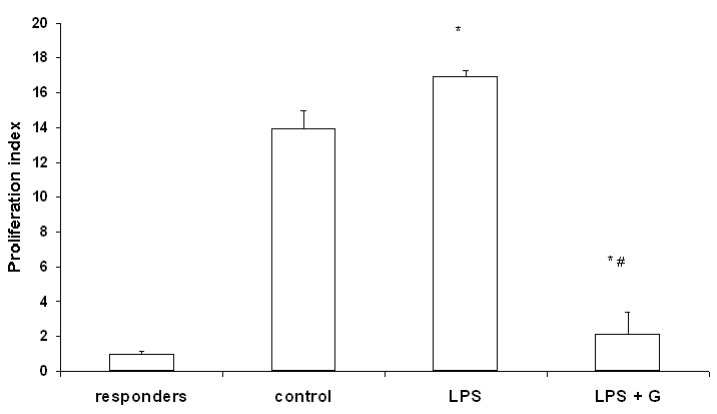
**Effect of alcoholic ginger extract on the antigen presenting function of murine peritoneal macrophages**. Peritoneal macrophages were incubated with LPS in the presence or absence of ginger extract for 24 h. These pre-treated macrophages were used as the sole source of APCs along with syngeneic T cells as the responder cells in the presence of allostimulation. T cell proliferation was measured by MTS assay after 72 h. incubation time. Results are presented as proliferation index equating the absorbance value (at 490 nm) of responder cells as 1. Responder cells only – no macrophages, Control- macrophages were not pre-treated, LPS- macrophages pre-treated with LPS only, LPS + G - macrophages pre-treated with LPS and ginger extract. Data represent mean ± S.D (n = 9). * *p *≤ 0.05 in comparison to Control. # *p *≤ 0.05 in comparison to LPS.

**Figure 5:**
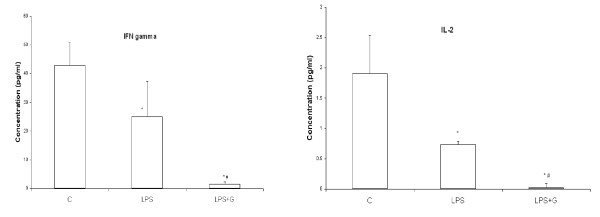
**Effect of alcoholic ginger extract on IL-2 (A) and IFN-γ (B) production by T cells in presence of alloantigen stimulation**. Peritoneal macrophages were incubated with LPS in the presence or absence of ginger extract for 24 h. These pre-treated macrophages were used as the sole source of APCs along with syngeneic T cells as the responder cells in the presence of allostimulation. IL-2 and IFN-γ production was measured in the culture supernates after 72 h. incubation period. * *p *≤ 0.05 in comparison to Control. # *p *≤ 0.05 in comparison to LPS.

A decreased expression of MHC class II and costimulatory molecules on macrophages by ginger extract suggests, that ginger extract treated macrophages are deficient in their role as APCs. To confirm the inhibitory effect of ginger extract on antigen presentation by macrophages, we performed a primary MLR where ginger extract treated macrophages were used as the only APC source. As shown in Figure 4, we observed that control macrophages as well as LPS treated macrophages induced T cell proliferation in response to alloantigen stimulation. However in case of macrophages treated with ginger extract in presence of LPS stimulation a significant decrease in T cell proliferation was observed. We also measured the production of IL-2 and IFN-γ in the supernatant of the primary MLR. A decrease in both IL-2 and IFN-γ production was observed in case of ginger extract treatment in presence of LPS stimulation (Fig. 5).

## Discussion

The aim of the present study was to determine the effect of ginger extract on macrophage activation and APC function. Our results indicate that ginger extract inhibits LPS induced activation of macrophages by inhibiting the proinflammatory cytokine and chemokine release and decreasing the costimulatory molecule expression. Our results support the previous studies that reported the suppressive effect of ginger extract on expression of pro-inflammatory genes [7]. It can be concluded from our data as well as the previous reports that ginger extract inhibits pro inflammatory cytokine release by macrophages irrespective of the monocyte heterogeneity.

Our results also indicate that GE inhibits macrophage activation leading to decrease in T cell proliferation in response to alloantigen. We observed an inhibition of proinflammatory cytokine release by ginger extract treated macrophages. In addition we observed a decrease in costimulatory molecule expression on ginger extract treated macrophages. Further ginger extract also decreased the MHC II expression on LPS activated macrophages. The functional consequence of the inhibitory effect of ginger extract on macrophages was seen in form of decrease in T cell proliferation in the primary MLR. This is further supported by decreased production of IL-2 and IFN-γ in the primary MLR containing ginger extract treated macrophages as APCs. Ginger is known to be anti-inflammatory in nature but its mechanism of action is not completely known. Our study for the first time shows the mechanism by which ginger exerts its anti inflammatory effects on macrophage and T cell activation. Ginger extract not only inhibits proinflammatory cytokine production by macrophages but also decreases IFN-γ production, the key pro inflammatory mediator, by T cells.

LPS is an important activating stimulus. LPS stimulation leads to an effective classical macrophage activation [12,13]. Classically activated macrophages are highly pro inflammatory and they secrete IL-12. In the presence of IL-12 a Th1 response predominates. Activated Th1 cells produce IFN-γ, which initiates a positive feedback loop by priming and activating more macrophages and by upregulating their MHC class II expression. IFN-γ also acts synergistically with TNF-α in macrophage activation initiating three different signalling arms viz., 1) FADD-dependent binding and activation of caspase-8, 2) activation of JNK pathway and 3) activation of NFκB. IL-1β also plays a role in macrophage activation [13,14]. Binding of IL-1β to IL-1R1 activates both MAPK-AP1 and I-κB kinase-NFκB pathways. Ginger extract exerts an inhibitory effect on the production of IL-12 thereby indirectly suppressing the production of IFN-γ and its positive feedback loop of macrophage activation. Inhibition of macrophage activation is further enhanced by a drastic decrease in TNF-α production along with inhibition of IL-1β production.

In addition to inhibition of macrophage activation ginger extract also negatively affects monocyte and leukocyte migration as evident from inhibition of RANTES and MCP-1 production. Increase RANTES production is associated with a wide range of inflammatory disorders. It acts by promoting leukocyte infiltration to the site of inflammation. At high concentrations RANTES induces activation of T cells in a manner not dissimilar to mitogens [15]. The activation of T cells by RANTES is followed by increased production of IL-2 and IFN-γ. Inhibition of RANTES production by ginger extract not only suppresses inflammation, but also restricts T cell activation, as observed by decreased IFN-γ production (Fig. 5). MCP-1 is a pro inflammatory cytokine responsible in the appearance of a monocyte rich inflammatory infiltrate [16]. Ginger extract inhibits the production of MCP-1 completely indicating an inhibition of macrophage activation.

The classically activated macrophages play a role as effector cells in Th1 cellular response by upregulating surface molecules like MHC class II and B7. Ginger extract down regulates the expression of both MHC class II and B7 molecules as shown in Figure 2 and 3. This in turn inhibits the activation of CD4+ T lymphocytes which is evident from decreased IFN-γ production (Fig. 5) and also inhibition of CD4+ T lymphocyte proliferation in response to alloantigen (Fig. 4).

The present study confirms the inhibitory effect of ginger extract on production of proinflammatory cytokines as shown by previous studies. In addition it demonstrates a novel inhibitory property of ginger. The inhibition of costimulatory molecule and MHC class II molecules by ginger extract suggests that ginger has inhibitory effect on macrophage maturation, activation as well as on its APC function. Antigen presentation by MHC class II molecules alongwith the costimulatory signal are both necessary to initiate an effective T cell activation. A decrease in the expression of either or both fails to bring about T cell activation followed by proliferation. We show an inhibitory effect of ginger on T cell proliferation which is an indirect effect mediated by inhibition of macrophage activation. Inhibition of T cell activation and proliferation is also indicated by a low IL-2 and IFN-γ production.

## Conclusion

In conclusion we found ginger, a known anti-inflammatory botanical, indeed has significant suppressive effect on both macrophage activation and T cell proliferation. Our data indicates this is due to a direct effect of ginger on macrophage activation and its APC function. Future studies will be directed towards elucidating the signal transduction pathways inhibited by ginger.

## Competing interests

The author(s) declare that they have no competing interests.

## Authors' contributions

ST contributed in acquisition, analysis and interpretation of the data.

DB contributed in data acquisition

DSK revised and critically improved the content of the manuscript.

All authors have read and approved the final version of the manuscript.

## Pre-publication history

The pre-publication history for this paper can be accessed here:

http://www.biomedcentral.com/1472-6882/8/1/prepub
